# Role of tangential biopsy in the diagnosis of nail psoriasis^☆^

**DOI:** 10.1016/j.abd.2023.11.005

**Published:** 2024-05-23

**Authors:** Laura Bertanha, Ingrid Iara Damas, Rafael Fantelli Stelini, Maria Letícia Cintra, Nilton Di Chiacchio

**Affiliations:** a Department of Pathological Anatomy, Medical Sciences College, Universidade Estadual de Campinas, Campinas, SP, Brazil; b Dermatology Service, Hospital do Servidor Público Municipal de São Paulo, São Paulo, SP, Brazil

**Keywords:** Biopsy, Histopathology, Nail diseases, Psoriasis

## Abstract

**Background:**

Histopathology can be crucial for diagnosis of inflammatory nail diseases. Longitudinal excision and punch biopsies are the most used techniques to obtain the tissue sample. However, there is a low clinical-histopathological correlation, besides the risk of nail dystrophy. Tangential excision biopsy (TB) is a well-established technique for the investigation of longitudinal melanonychia. TB could also be used to evaluate diseases in which histopathological changes are superficial, as in psoriasis.

**Objective:**

To study the value of TB in the histopathological diagnosis of nail psoriasis.

**Methods:**

This is a prospective and descriptive study of the clinical-histopathological findings of samples from the nail bed or matrix and nail plate of 13 patients with clinical suspicion of nail psoriasis. Biopsies were obtained through partial nail avulsion and TB.

**Results:**

In nine patients, the hypothesis of psoriasis was confirmed by histopathology; in one, the criteria for diagnosing nail lichen planus were fulfilled. The tissue sample of only one patient did not reach the dermal papillae, and, in four of 13 patients, the adventitial dermis was not sampled. No patient developed onychodystrophy after the procedure.

**Study limitations:**

In three patients, the clinical and, consequently, histopathological nail changes were subtle. Also, in one patient’s TB didn’t sample the dermal papillae.

**Conclusions:**

TB is a good option to assist in the histopathological diagnosis of nail psoriasis, especially when appropriate clinical elements are combined. Using this technique, larger and thinner samples, short postoperative recovery time, and low risk of onychodystrophy are obtained.

## Introduction

Clinical changes in nail disorders are not only limited but also are shared by different types of processes. Onycholysis, for example, can be a manifestation of different diseases such as lichen planus, onychomycosis, or nail psoriasis.^1^ In the case of suspected inflammatory diseases of the nails, without associated skin involvement, a nail biopsy is necessary to assist in accurate diagnosis and specific treatment.^1^

Involvement of the nail matrix in psoriasis manifests as nail plate pitting, thinning, onychorrhexis, red dots on the lunula, and true leukonychia. In the nail bed, the 'oil spot’ or 'salmon stain' changes, subungual hyperkeratosis, onycholysis, and splinter hemorrhages can be identified.^1^ Pittings, subungual hyperkeratosis, and onycholysis are the most common findings.^2^

The clinical diagnosis can be corroborated using a dermatoscope, enabling clearer identification of the erythematous halo next to onycholysis. It also highlights dilated, elongated, and tortuous capillaries in hyponychium and nail bed, in addition to compact subungual hyperkeratosis on the free edge.^3^

Histopathological changes in nail psoriasis are limited to the nail plate or horny layer, epidermis, papillary dermis, and sparing the reticular dermis.^1^ As in the skin, there is an influx of neutrophils (considered a major criterion for the diagnosis of nail psoriasis), psoriasiform hyperplasia of the epidermis, dilated and tortuous venules filling the dermal papillae, and perivascular lymphoid infiltrate in the underlying adventitial dermis.^1^ Focal parakeratosis can form in the dorsal, intermediate or ventral portions of the nail plate, producing pitting, true leukonychia, or onycholysis, respectively. When the entire matrix is affected, there may be marked dystrophy.^4^

However, certain characteristics are distinct in the nail unit: while in the hyponychium, there is a loss of the granulosa layer normally present; in the matrix and nail bed there is hypergranulosis. Spongiosis may occur, forming prominent serum crusts on papillae top, due to hypervascularization, increased vascular permeability in the dermal papillae, and blockage of exudate elimination caused by apposition of the nail. In addition, hemorrhage spots in the granulosa and corneal layer can be seen.^4^, ^5^

As onychomycosis and psoriasis share some histological features, Grover et al. recommended that negative periodic acid-schiff immunohistochemical (PAS) reactions for fungi should be included as an additional diagnostic criterion.^5^, ^6^

Longitudinal and 3-mm punch biopsies are currently the most used techniques for the investigation of nail disorders. In the longitudinal technique, one incision is made through the nail bed, the entire matrix, and the proximal nail fold. The second incision parallel to the initial one, no more than 3-mm from the first, is made in the lateral nail fold. The tissue between the two incisions is excised from the underlying bone and the defect is then sutured.^1^, ^5^ Expected complications include reduction in nail width, malalignment of the axis of the regrowing nail, scarring of the nail bed, onycholysis, or growth of nail spicules.^7^

A punch biopsy can be performed in any area of the nail apparatus. Partial avulsion of the nail plate may or may not be necessary. The 3-mm punch is used, rotating it down to the bone, and the base of the fragment is cut with sharp iris scissors. The resulting defect does not require suturing. In the bed region, biopsy collection rarely generates dystrophy, but it can result in onycholysis. In the proximal matrix, there is a risk of onychodystrophy.^1^, ^7^

In both techniques, onycholysis and nail dystrophy may appear.^1^, ^7^ Incidentally, low clinicopathological correlations may occur.^5^, ^6^ The tangential excision (TB) biopsy technique is well-known for diagnosing longitudinal melanonychia. Former studies have demonstrated that the thickness obtained is sufficient for the differential diagnosis of the various processes that manifest with melanonychia and the morbidity is lower, compared to more invasive techniques.^8^, ^9^, ^10^ There are two advantages of using TB. First, with TB, it is possible to obtain a sample with a width greater than that of 3-mm, allowing for an increased number of tissue sections. This could aid in the histopathological evaluation of inflammatory diseases characterized by superficial histopathological changes, such as psoriasis. Second, by using the TB technique, deep tissue is spared, reducing the risk of cicatricial onychodystrophies. The authors aimed to evaluate the results of the nail tangential excision biopsy technique for the histopathological diagnosis of psoriasis.

## Methods

This is prospective descriptive work. The study proceeded after approval from the Institutional Research Ethics Committee (CAEE: 55474521.1.0000.5404). Between 2021 and 2022, 13 patients with a diagnosis hypothesis of nail psoriasis were selected during the nail disease clinic care. The following nail changes were considered: onycholysis, splinter hemorrhages, subungual hyperkeratosis, salmon patches/oil spots, and pitting. Signed informed consent forms included the risk of onychodystrophy.

**Figure 1:**
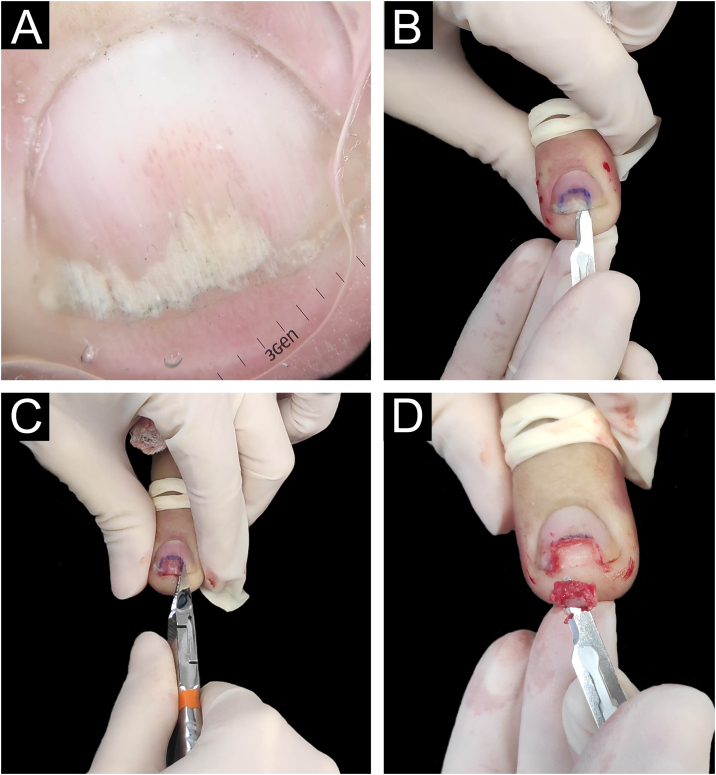
Longitudinal tangential biopsy technique – (A) Onychoscopy: regular onycholysis with salmon patch and dilated linear vessels; (B) Partial detachment of the nail plate with a number 15 scalpel blade, in the demarcated region; (C) Partial cutting of the plate with pliers; (D) Slightly thicker tangential biopsy of the nail bed (sample measuring 0.7 × 0.2 × 0.1 cm).

Biopsies were obtained from the nail with the most prominent involvement, preferably from the nail bed. The same nail specialist and dermatologist performed the procedures. Aseptic surgical technique with distal block anesthesia without vasoconstrictor and tourniquet were used. The nail plate was detached using a number 15 scalpel blade to maintain the integrity of the epithelium at the time of nail avulsion. Then, the nail was partially cut with pliers, and tangential excision was performed on the nail bed or matrix (Fig. 1). Finally, the tourniquet was removed, and the occlusive dressing was applied. A representative diagram of the nail complex was drawn on filter paper, on which the removed specimen was placed, in the location corresponding to its removal, with the epithelium facing upwards. This procedure prevents the laminar sample from curling during fixation and improves histopathological analysis.^11^

Then, the paper was folded to wrap the material, it was stapled, and immersed in 10% buffered formaldehyde. The removed nail plate was placed in a dry polypropylene tube (Eppendorf). Both samples were sent to histological processing at the Pathology Department. In the laboratory, the sample was removed from the filter paper and placed inside a groove produced in a thin slice of *Solanum tuberosum* (English potato), to assure proper histological inclusion. Both (potato and surgical material) were dehydrated, diaphanized, and embedded in paraffin, as a single material, and were subsequently paraffin-embedded, cut at various levels, and stained.^12^ The nail plate was softened with potash, washed, cut, fixed, and processed for histological analysis. The patients were instructed on how to perform local cleaning 48 hours after the procedure and to use analgesics and systemic antibiotics for seven days. There was clinical follow-up for a minimum period of 6 to 12 months, until complete nail growth.

The specimens were blindly analyzed by one dermatopathologist and one dermatologist. The following variables were evaluated: subungual hyperparakeratosis, serous and/or neutrophilic exudate, and hemorrhage in the stratum corneum, hypo-/hypergranulosis, papillomatosis, spongiosis, hyperplasia of the epidermis, dilated dermal papillae vessels, type and location of the inflammatory infiltrate in the papillary or adventitial dermis. A search for fungi was carried out in all samples using PAS-stain.

## Results

**Figure 2:**
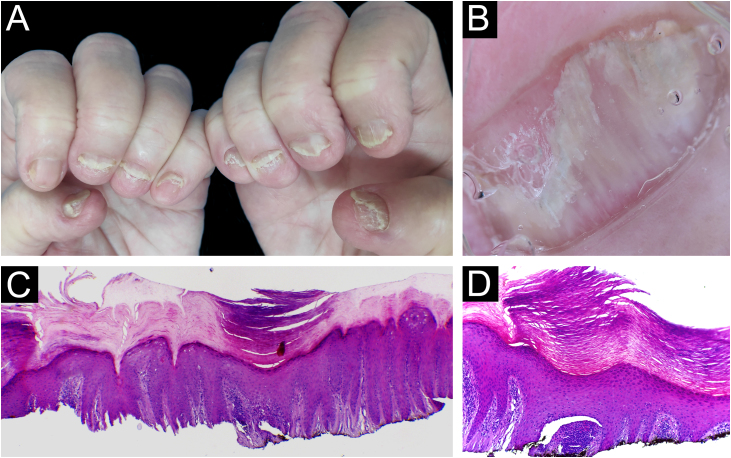
Lichen planus ‒ (A) All fingernails affected: regular onycholysis, subungual hyperkeratosis and onychodystrophy; (B) Onychoscopy: red spots on the lunula, subungual hyperkeratosis and irregular striated leukonychia; (C) Nail bed with significant lymphoid infiltrate within the dermal papillae; (D) lichenoid lymphoid infiltrate fills the papilla, with a subepidermal cleft. Hematoxilina & eosin, ×100 (C) and × 400 (D).

Biopsies were obtained from twelve fingers and one toe of 13 different patients (five men and eight women). One of the samples presented unequivocal findings of nail lichen planus (lichenoid lymphoid infiltrate, with dermo-epidermal cleft and absence of dilated dermal papillae vessels). Clinically, there was dystrophy of all hand nails, with onycholysis, subungual hyperkeratosis, splinter hemorrhages, red spots on the nail bed and lunula, and longitudinal striae (Fig. 2).

**Figure 3:**
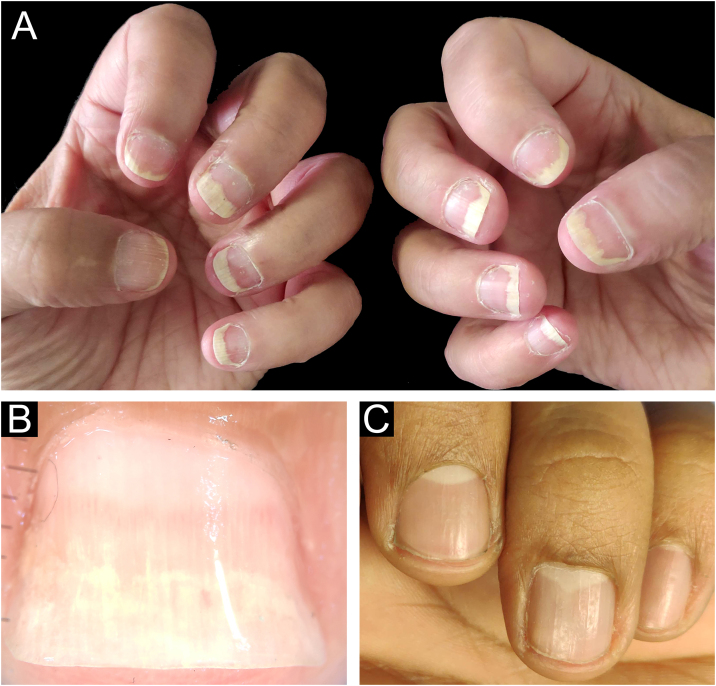
Nail psoriasis ‒ (A) Regular onycholysis with salmon patch on several digits; (B) Onychoscopy: regular onycholysis with salmon patch; (C) Coarse nail pittings.

**Figure 4:**
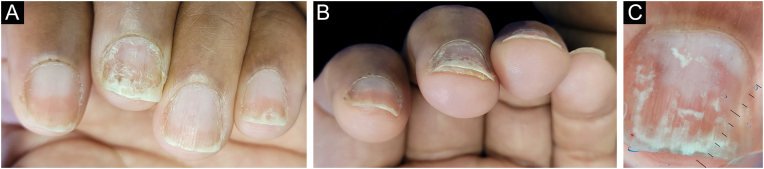
Nail psoriasis ‒ (A) Regular onycholysis with salmon patch on several digits; (B) Compact subungual hyperkeratosis, seen on the free nail edge; (C) Onychoscopy: regular onycholysis with salmon patch and punctate leukonychia.

The main clinical features of the remaining 12 samples were onycholysis (9/12), splinter hemorrhages (9/12), oil spot (8/12), salmon patch (7/12), pitting (7/12) and subungual hyperkeratosis (5/12). Only one of the 12 patients had pitting as an isolated change so TB was obtained from the nail matrix. The others had nail changes predominantly related to nail bed (Figure 3, Figure 4).

Nail matrix TB allowed the identification of vascular ectasia within papillary dermis, although other histological features of psoriasis were missing.

Five patients concomitantly had psoriasis skin lesions. Of these, three showed discrete nail changes, only one presented a salmon patch with onycholysis, one presented subungual hyperkeratosis with splinter hemorrhages, and one presented isolated pitting. In these three, the histological changes of TB and nail plate were insufficient to meet the criteria for a diagnosis of psoriasis. The TB samples' width ranged from 1.1 cm to 0.3 cm in width, which were equal to or greater than those that would be obtained using the 3-mm punch or longitudinal biopsies. Still, their thickness ranged from 0.1 to 0.15 cm.

**Figure 5:**
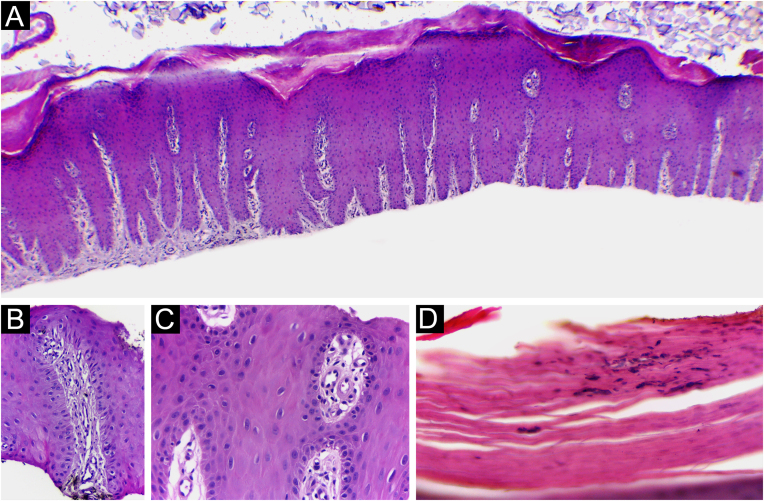
Psoriasis (nail bed) ‒ (A) Psoriasiform hyperplasia of the epidermis, hypervascularized papillary dermis and thinning of the suprapapillary stratum spinosum; (B and C) Thinning of the suprapapillary stratum spinosum, highly vascularized papillae and absence of spongiosis; (D) Parakeratosis and intracorneal neutrophils exudate. Hematoxilina & eosin, 40 (A) and ×400 (B‒D).

In four out of 13 patients, the adventitial dermis was not taken and, in one patient, neither was the papilla. The histological findings in the 12 samples were (Fig. 5): absence of spongiosis (12/12), presence of dilated papillae vessels (11/11), parakeratosis (11/12), regular epidermal hyperplasia of the epidermis (9/12), hypogranulosis focal (5/12), thinning of the epidermis in the suprapapillary region (5/12), Munro’s microabscess (4/12) and focal hypergranulosis (3/12). Sending the nail plate was important for fungal research. In one case, it was possible to identify, in one focus, a small number of septate filamentous fungi. The presence of septate filamentous fungi in nail plates could be attributed to a primary fungal infection or secondary fungal infection of psoriasis. Psoriasis and dermatophytosis have a lot of common histopathological findings. However, the presence of parakeratosis on the papillae, hypogranulosis, thinning of the suprapapillary epidermis, and increased density of dermal vessels allowed diagnostic confirmation of concomitant psoriasis.

**Figure 6:**
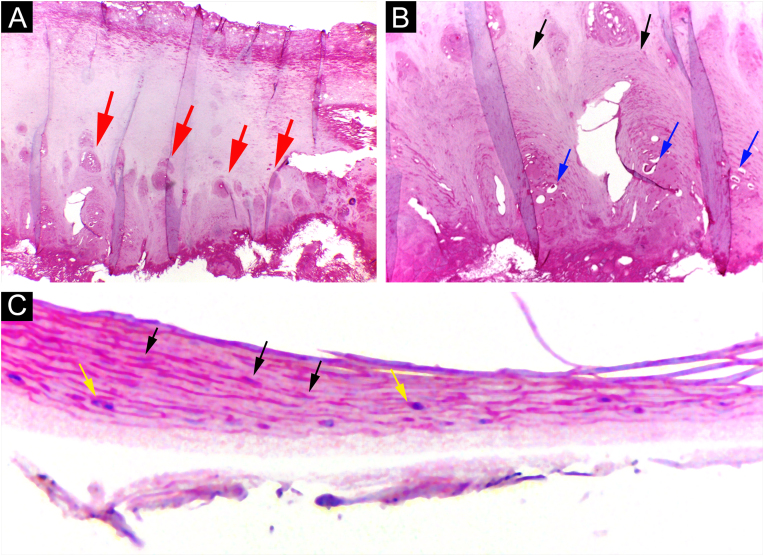
Psoriasis (nail plate) ‒ (A) Papillomatosis (the tops of the papillae superficialized from the bed – red arrows); (B) Intracorneal serous exudate on the top of the papillae (blue arrows), with parakeratosis (black arrows); (C) Keratinocyte nuclei (parakeratosis ‒ black arrows) and rare neutrophils (yellow arrows). Hematoxilina & eosin, ×100 (A, B) and ×400 (C).

Through nail plate histopathological evaluation, additional findings suggestive of psoriasis were recorded, such as the top of the superficialized papillae, suprapapillary intracorneal serous exudate, parakeratosis, and intracorneal neutrophils. Assessment of the nail plate contributed to the final diagnosis in three out of 12 cases (Fig. 6).

In summary, by obtaining the 13 TB cases, it was possible to define the diagnosis of lichen planus in one and psoriasis in 9. In the remaining 3, sufficient criteria were not met to achieve the diagnosis.

**Figure 7:**
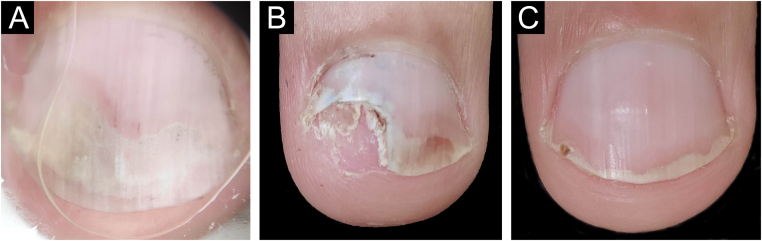
Nail psoriasis -before and after tangential biopsy ‒ (A) Onychoscopy of the area to be biopsied- regular onycholysis with salmon patches and splinter hemorrhages; (B) 7-day postoperative period ‒ excellent bed healing; (C) 30-days post-operative period – complete nail recovery.

**Figure 8:**
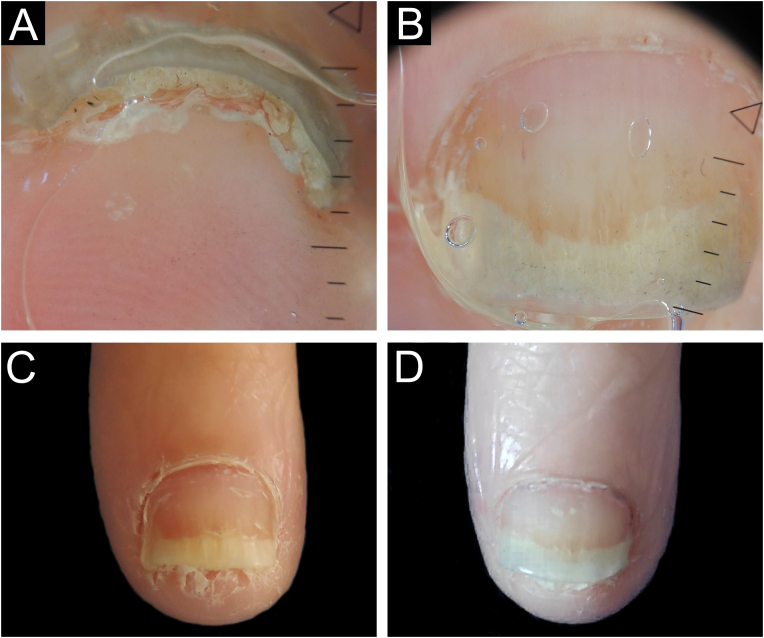
Nail psoriasis -before and after tangential biopsy ‒ (A and B) Onychoscopy of the area to be biopsied - regular onycholysis with oil spot, splinter hemorrhages and compact subungual hyperkeratosis; (C) Immediate pre-operative period; (D) 30-days post-operative – complete nail recovery.

The time to resume daily activities was up to seven days. Nail dystrophy was not observed in any patient after six months to one year of follow-up (Figure 7, Figure 8).

## Discussion

In the approach to inflammatory nail diseases, obtaining a biopsy is extremely important for defining the diagnosis, planning treatment, and estimating the prognosis. However, dermatologists are reluctant to biopsy the nail, due to the risk of dystrophies, lack of prior training in nail surgery and, not rarely, inconclusive histopathological findings.^5^, ^6^, ^13^, ^14^, ^15^ In 1999, Haneke demonstrated the efficacy and safety of TB for the diagnosis and treatment of nail melanonychia, avoiding permanent onychodystrophies.^8^ Di Chiacchio et al. reported that with this technique it is possible to obtain specimens with sufficient thickness for differential diagnosis between hypermelanosis, lentigo, junctional or compound nevus, and in situ melanoma, as all the histopathological changes are restricted to the epidermis or upper part of the dermis. They also found that the procedure induced minimal damage to the matrix, with excellent aesthetic results.^10^ Since then, the use of this technique remained restricted to the elucidation of melanonychia diagnosis. In psoriasis and lichen planus, the main inflammatory nail diseases, histological changes are seen in the superficial layers of the nail bed/matrix. Therefore, the possibility of also using TB in the diagnosis of these diseases may encourage dermatologists to collect more biopsies and, consequently, define the diagnosis in a greater number of patients.

**Figure 9:**
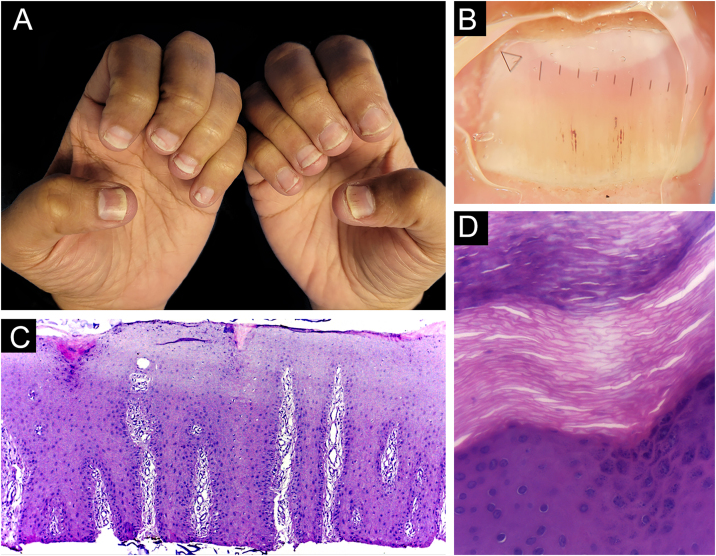
Nail psoriasis ‒ (A) All fingernails with regular onycholysis, salmon patch, some oil spots, subungual hyperkeratosis and splinter hemorrhages; (B) Onychoscopy: distal leukonychia and splinter hemorrhages; (C) High papillae vascular density; (D) Area of hypogranulosis, on the left, and area of hypergranulosis, on the right. Hematoxilina & eosin ×100 (C) and ×400 (D).

**Figure 10:**
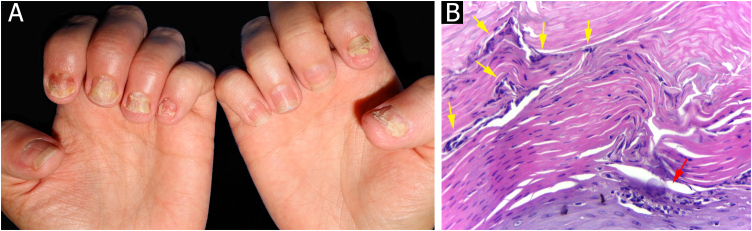
Nail psoriasis ‒ (A) Regular onycholysis on all fingernails, some with oil spots, subungual hyperkeratosis, and total dystrophy; (B) Neutrophilis exudate amidst superficial keratinocytes (red arrow); neutrophils within parakeratotic scale (yellow arrows). Hematoxilina & eosin, ×400.

Punch biopsy, without prior avulsion of the plate, would be the ideal technique indicated for the investigation of inflammatory nail diseases, due to better preservation of the superficial tissue morphology. However, it is a difficult task to introduce the punch through the nail plate, maintaining the adhesion of the plate to the bed/matrix during the rotation of the instrument. Shearing often occurs. Furthermore, the idea is to soften the nail plate by using alkali before fixation, to optimize the histological preparations. This procedure and the delay in fixing the specimen can damage elements of the nail bed/matrix, which would help to define the diagnosis.^13^, ^14^, ^15^ Careful detachment of the nail plate with a scalpel blade, in TB, proved to be effective in maintaining the integrity of the epithelium. In the present study, sending the overlying plate separately allowed additional microscopic evaluation, without interfering with the histopathological processing. The most frequent histomorphological findings in these samples were: the absence of spongiosis (12/12), dilated papillae vessels (11/11), and parakeratosis (11/12) (Figure 5, Figure 9, Figure 10). Grover et al. highlighted the findings of hyperkeratosis with parakeratotic areas (91%), and dilated vessels, in all patients with a confirmed clinical diagnosis of psoriasis.^6^

The authors found full clinical-histological correlation in 75% (9/12) of patients, a higher rate than those already reported, whether in samples obtained by longitudinal or punch biopsies.^5^, ^6^ Hanno et al. detected histomorphological findings that met the criteria for a specific nosological diagnosis in only 40% of the studied cases; of the six patients with clinical suspicion of psoriasis and the seven suggestive of lichen planus, in only four and three patients, respectively, the diagnoses were confirmed.^5^ Grover et al. studied 22 patients with a clinical diagnosis of nail psoriasis, collected samples through longitudinal and punch techniques, and achieved a clinicopathological correlation of 54%.^6^ Perhaps the highest rate of clinical-histological correlation in the studied patients was due to the greater sampling in the width of the excised tissue, made possible by TB, associated with the care used in the technique, from sample collection to histological reading, in addition to the inclusion of additional histological criteria.

Hanno et al. (1986) proposed the histological criteria to be considered for diagnosis of nail psoriasis.^5^ Hyperkeratosis, with parakeratosis, intracorneal serous exudate, a granular layer of irregular thickness, and psoriasiform hyperplasia of the epidermis of the nail bed, with dilation of the papillary blood vessels were described as minor criteria. The finding of neutrophils on the bed epithelium surface, with parakeratotic scales adhered to the nail plate, was considered the major criterion. To confirm the diagnosis of nail psoriasis, the major criterion would be necessary, with or without an associated minor criterion.^5^ In 2005, Grover et al. proposed an additional criterion: negative PAS-stain for fungi.^6^ However, the concomitance of nail psoriasis and onychomycosis is possible and described.^16^ Indeed, neutrophil aggregates seem to be more common in onychomycosis and parakeratosis, and more frequent in psoriasis.^17^ The authors postulate that the value neutrophils exudate as a major and mandatory criterion for diagnosis, may be the main obstacle to the best clinicopathological correlation of former studies. In the samples the authors analyzed, a good clinical-histological correlation was obtained when using the following criteria:

- Mandatory criterion: dilatated dermal papillae vessels (Fig. 5);
- Secondary criteria (at least three): absence of spongiosis, presence of parakeratosis, psoriasiform epidermal hyperplasia, focal thinning of the granular layer and suprapapillary epidermis, and intra- or subcorneal neutrophils exudate (Figure 5, Figure 9, Figure 10).

Additional nail plate analysis supported a diagnosis of psoriasis in three of 12 cases, and in six cases, the analysis of the subungual tissue was essential for the diagnosis. Thus, only a nail clipping specimen would be insufficient for diagnosis in the majority of patients. Even so, the authors emphasize that the nail plate must be analyzed, as it can provide additional information, in addition to allowing fungal research (Fig. 6).

If the clinical findings are subtle, with discreet pitting, salmon patch, or isolated subungual hyperkeratosis, a histological analysis may be inconclusive. In these cases, the authors suggest clinical follow-up and biopsy indication if there are other associated clinical signs.

In patients with subungual hyperkeratosis, the TB of the nail bed must be a little deeper, due to the risk of not reaching the dermis. In the matrix, a superficial tangential biopsy is sufficient to obtain adequate sampling of the papillary dermis. Since many patients with nail psoriasis present with mixed clinical findings of nail bed and matrix involvement, it is preferable and sufficient to obtain a biopsy of the nail bed.

## Limitations

In three of the 12 included patients, the clinical and, consequently, histological nail changes were subtle (one of them only had a salmon patch with onycholysis, another subungual hyperkeratosis with splinter hemorrhages, and the last, isolated pitting). Also, in one patient TB sample, the dermal papilla was not taken, although sufficient histological changes were found in the epidermis and nail plate to reach a psoriasis diagnosis.

## Conclusion

The TB technique was found to be a good option for diagnosing the main inflammatory nail diseases because, through it, larger and thinner samples, short postoperative recovery time, and low risk of onychodystrophy were achieved. TB allowed for defining the diagnosis, especially when sufficient clinical elements were associated with histomorphological findings.

## Financial support

This work was carried out with the support of the Coordination for the Improvement of Higher Education Personnel - Brazil (CAPES) - Financing code 001.

## Authors’ contributions

Laura Bertanha: Participated in generating and analyzing the data; wrote the majority of the original draft of the paper, reviewed the pertinent raw data on which the results and conclusions of this study are based and approved the final version of this paper.

Ingrid Iara Damas: Participated in generating data, writing the paper and approved the final version of this paper.

Rafael Fantelli Stelini: Participated in generating data, writing the paper, reviewed the pertinent raw data on which the results and conclusions of this study are based and approved the final version of this paper.

Maria Letícia Cintra: Participated in designing, generating and analyzing the data; wrote the paper and reviewed the pertinent raw data on which the results and conclusions of this study are based and approved the final version of this paper.

Nilton Di Chiacchio: Participated in generating data, writing the paper, reviewed the pertinent raw data on which the results and conclusions of this study are based and approved the final version of this paper.

## Conflicts of interest

None declared.
